# T Wave Oversensing

**Published:** 2010-06-05

**Authors:** Johnson Francis

**Affiliations:** Malabar Institute of Medical Sciences, Calicut, Kerala, India.

**Keywords:** Pacemaker, T Wave Oversensing, Implantable cardioverter defibrillators

T wave oversensing is quite a familiar phenomenon to anyone who has watched cardiac monitors for sufficiently long periods in the coronary care unit. The counter starts giving dual beeps for every cardiac cycle instead of one. If it persists during several cycles, the displayed rate goes up and the monitor sounds an alarm indicating 'tachycardia'. This occurs usually when the monitoring lead displays a tall T wave which is also sharp. R wave sensing algorithms of the devices do not sense T wave because the slew rate of the T wave is much less than that of the R wave. But slew rate of T waves may change with time and also depending on parameters like potassium levels and ischemic events. Just as the external cardiac monitor, implantable cardiac devices are also prone for T wave oversensing though they are meant to detect only R waves. This can lead to a variety of problems in the functioning of the devices.

In the conventional pacemakers, T wave oversensing causes inhibition of output leading to bradycardia or asystole. Implantable cardioverter defibrillators (ICD) detect these as tachyarryhthmic events and may either give overdrive pacing or defibrillator shocks depending on the programmed parameters. The tall T waves in short QT syndrome is prone to cause oversensing. It is possible to overcome T wave oversensing by reprogramming the device [1]. T wave oversensing has also been reported in long QT syndrome and in one case an epicardial pace/sense lead with stable R waves was needed to resolve the problem due to T wave oversensing [2]. Silver JS et al used a novel strategy to eliminate inappropriate shocks due to T wave oversensing in a biventricular ICD. This was achieved by using a V-V pace delay which meant sequential rather than simultaneous biventricular pacing to avoid T wave oversensing without reducing the tachyarrhythmia detection sensitivity [3]. An interesting case of T wave oversensing during exercise in a patient implanted with an ICD for Brugada syndrome has also been documented [4]. Sometimes T wave oversensing can be demonstrated by exercise treadmill testing [5]. Dynamic temporal changes in the electrocardiogram in Brugada syndrome make them vulnerable for T wave oversensing [6,7]. A persistent or transient lowering of the R wave amplitude can also lead to T wave oversensing [8].

Changing the leads and reprogramming may not be sufficient in certain cases. In one such case, implantation of a new device with automatic sensitivity control was useful in eliminating T wave oversensing and inappropriate ICD shocks  [5]. Proper filtering of the detected signals is also thought to be important in preventing inappropriate shocks due to T wave oversensisng [9]. T wave oversensing can occur without any appreciable change in the surface ECG. This is because changes in endocardially recorded electrograms may not be reflected in surface ECG. An unfavourable R to T wave amplitude from the endocardial lead can lead to double counting  [10]. Cardiac sarcoidosis can cause decreased R wave amplitudes and inappropriate defibrillator shocks due to T wave oversensing [11].

In this issue of the journal Frutos M et al [12] report a novel case in which T wave oversensing leading to inappropriate antitachycardia pacing was detected by remote monitoring. The problem was corrected by calling back the patient and reprogramming the device. Partial inhibition of antitachycardia pacing due to T wave oversensing is another interesting phenomenon. Intermittent T wave oversensing was the cause of this partial inhibition. The problem was solved with an ingenious method of reprogramming the paced ventricular blanking period to a higher level [13].

T wave oversensing may lead to loss of biventricular pacing [14,15]. This can be an important reason for lack of improvement or worsening functional status with biventricular pacing. Transient loss of biventricular pacing due to oversensing of tall T waves due to hyperkalemia in a patient undergoing dialysis has also been noted. This patient also had inappropriate ICD shocks due to the same reason. Oversensing was abolished by lowering the potassium levels in the dialysis fluid [16]. Similar situation of consistent T wave oversensing during hyperglycemia, responding to treatment of diabetes has also been reported[17].

Inappropriate pacing due T wave oversensing in an ICD recepient has been documented in literature. In this case, intermittent T wave oversensing invoked the ventricular rate stabilization algorithm causing inappropriate VVI pacing. Maximum sensitivity was reduced from 0.3 mV to 0.45 mV to solve this problem [18].
